# Researchers’ participation in and motivations for engaging with research information management systems

**DOI:** 10.1371/journal.pone.0193459

**Published:** 2018-02-23

**Authors:** Besiki Stvilia, Shuheng Wu, Dong Joon Lee

**Affiliations:** 1 School of Information, Florida State University, Tallahassee, Florida, United States of America; 2 Graduate School of Library and Information Studies, Queens College, Queens, New York, United States of America; 3 University Libraries, Texas A&M University, College Station, Texas, United States of America; Institut Català de Paleoecologia Humana i Evolució Social (IPHES), SPAIN

## Abstract

**Researchers’ participation in online RIMSs:**

This article examined how researchers participated in research information management systems (RIMSs), their motivations for participation, and their priorities for those motivations. Profile maintenance, question-answering, and endorsement activities were used to define three cumulatively increasing levels of participation: Readers, Record Managers, and Community Members. Junior researchers were more engaged in RIMSs than were senior researchers. Postdocs had significantly higher odds of endorsing other researchers for skills and being categorized as Community Members than did full and associate professors. Assistant professors were significantly more likely to be Record Managers than were members of any other seniority categories. Finally, researchers from the life sciences showed a significantly higher propensity for being Community Members than Readers and Record Managers when compared with researchers from engineering and the physical sciences, respectively.

**Researchers’ motivations to participate in RIMSs:**

When performing activities, researchers were motivated by the desire to share scholarship, feel competent, experience a sense of enjoyment, improve their status, and build ties with other members of the community. Moreover, when researchers performed activities that directly benefited other members of a RIMS, they assigned higher priorities to intrinsic motivations, such as perceived self-efficacy, enjoyment, and building community ties. Researchers at different stages of their academic careers and disciplines ranked some of the motivations for engaging with RIMSs differently. The general model of research participation in RIMSs; the relationships among RIMS activities; the motivation scales for activities; and the activity, seniority, and discipline-specific priorities for the motivations developed by this study provide the foundation for a framework for researcher participation in RIMSs. This framework can be used by RIMSs and institutional repositories to develop tools and design mechanisms to increase researchers’ engagement in RIMSs.

## Introduction

Services that offer reliable and scalable determination and disambiguation of research identities are essential services that data repositories and digital libraries need to provide. Such services enable distributed grouping, linking, aggregation, and retrieval of scholarship; evaluation of the research productivity and impact of individuals, groups, and institutions; and the identification of expertise [1–3]. Publishers, libraries, universities, search engines, and content aggregators use many different research identity management systems, often referred to as research information management systems (RIMSs) or current research information systems (CRIS), with different data models, levels of coverage, and levels of quality (e.g., Florida ExpertNet, Google Scholar, ORCID, REACH NC, ResearchGate). These databases use different approaches to and mechanisms for curating research identity information: manual curation by information professionals or users, including the subjects of identity data; automated data mining and curation scripts (aka bots); or some combination thereof. With universities engaging in the curation of digital scholarship produced by their faculty, staff, and students through institutional repositories (IR), some of these universities and IRs try to manage the research identity profiles of their contributors locally (e.g., Stanford Profiles). Some large academic libraries use the VIVO ontology [4] to make their data, including researcher identity information, discoverable and linkable for cross-institutional retrieval, processing, and analysis by both human and computational agents. The use of ontologies and Semantic Web technologies can make data machine-processable and “understandable,” and hence may reduce the cost of data aggregation and analysis. Ultimately, however, the completeness and accuracy of the data are what make RIMSs reliable. Although knowledge curation by professionals usually produces the highest quality results, it is costly and may not be scalable [5]. Libraries and IRs may not have sufficient resources and expertise to control the quality of large-scale uncontrolled metadata, often batch harvested and ingested from faculty-authored websites and journal databases [5]. The effective aggregation of data may require knowing community, discipline-based, and cultural differences in data and metadata quality requirements, rules, norms, and reference sources. They may need the participation of subject specialists, librarians, and especially the researchers themselves in data curation activities to ensure the quality and reliability of research identity data [3, 6–8].

The literature on online communities shows that successful peer-curation communities, those able to attract and retain enough participants, can provide scalable knowledge-curation solutions comparable in quality to the quality of professionally curated content [9]. Hence, the success of online research identity management systems may depend on the number of contributors and users they are able to recruit, motivate, and engage in research identity data curation. A significant body of research exists on what makes peer knowledge-creation and knowledge-curation groups and communities successful. Some of the issues and factors that may affect the success of peer curation of knowledge are peer motivations to contribute, the effectiveness of work articulation and coordination, task routing, and quality control (e.g., [10–12]). Researchers’ participation in the curation of research identity data has typically not been investigated, however. Furthermore, people may value different motivations at different levels [13]. Therefore, it is important to identify the value structure underlying researchers’ motivations for maintaining and sharing their research identity data. This article addresses the need for a greater understanding of how to design scalable solutions for research information management by examining how researchers participate in RIMSs, their motivations for participation, and the value structures underlying those motivations. We investigated the following research questions:

1. What are the types of researcher participation in online RIMSs?
2. What are researchers’ motivations for participating in RIMSs?

## Background

### Community participation

Roles are important in understanding member participation in an online community and in explicating and organizing the community’s work. Community roles can be formally defined and assigned together with related activities and power privileges, or they can be undertaken informally through self-selection [12,14]. Users may play different roles at the same time or at different times. Preece and Shneiderman [15] proposed a conceptual framework to characterize users’ successive levels of participation or roles in online social communities as evolving from reader to contributor, to collaborator, and finally to leader. Furthermore, users may abandon a community if it no longer meets their needs and objectives [12,15,16]. Wu and colleagues [17] found that researchers’ engagement in online RIMSs changed as their career status changed and influenced their perception of the costs and benefits of using a specific RIMS. Some researchers gave up curating their RIMS profile and actively promoting their research through the RIMS after they secured jobs and were no longer motivated by the objectives of those activities. Haustein and Larivière [18] analyzed journal articles from four disciplines (i.e., biomedical research, clinical medicine, health, and psychology) in Mendeley and found that the majority of Mendeley users were junior researchers, such as doctoral students, postgraduate students, and postdoctoral researchers (aka postdocs).

Participation in RIMSs may also vary by discipline. Thelwall and Kousha [19] examined the coverage of Scopus-indexed articles with DOI identifiers in ResearchGate and found that the arts, humanities, and some areas of social sciences were highly underrepresented relative to other disciplines. Mas-Bleda and colleagues [20], who studied the online presence of highly cited researchers working at European institutions, found that an online presence in a RIMS was higher among researchers in the social sciences, engineering, and health sciences than among those in the life sciences and physical sciences.

### Motivations to participate

Preece and Shneiderman [15] conducted a literature review to identify possible motivations for different levels of participation in online communities. For example, they found that a welcoming environment, safety, privacy, support for newcomers, encouragement by individuals they trusted or respected, contacts to answer their questions, and frequently updated and well-organized content were some factors that motivated readers. The ease of making contributions, recognition of the quality and quantity of their contributions, and gaining visibility or awards for their contributions were some factors that encouraged contributors. Trust and empathy that promoted belonging to the community, altruism, and the desire to build a reputation were some factors that encouraged collaborators.

The literature on online communities has shown that volunteer knowledge curators in open peer-production systems such as Wikipedia are mostly driven by intrinsic motivations, such as their interests in specific areas, which are often shaped by their organizational and ethnic affiliations, hobbies, professional experiences and expertise, and beliefs [11,12]. For example, Nov [11] found that enjoyment or having fun was the top motivation for contributing to English Wikipedia. It is noteworthy that these intrinsic motivations could produce both constructive and disruptive behaviors (i.e., trolling and vandalism) toward the community’s objectives [21].

In addition to Wikipedia, researchers examined users’ motivations for contributing to other online communities. Nov and colleagues [22] found a positive relationship between the motivation to build a reputation in the community and the number of tags contributed in Flickr. Similarly, in an earlier study examining an online network of legal professionals, Wasko and Faraj [23] found a significant positive effect on the motivation to build a reputation from the quality and volume of knowledge contributed. Hars and Ou [24] examined the motivations of contributors to free and open source software (FOSS) projects. They identified two categories of motivations: internal and external. Internal motivations included factors related to self-determination, as well as altruism and community identification. The external motivation category included factors related to future rewards (e.g., enhanced status and self-efficacy) and personal needs for open source software functionalities. As motivations to participate in FOSS projects, Lakhani and Wolf [25] identified self-identification with a community and a sense of obligation to contribute, along with the motivations of a sense of enjoyment and receiving rewards.

Previous studies have also examined motivations for specific types of knowledge-sharing activities, such as question answering. For instance, Raban and Harper [26] conducted a literature analysis and identified 14 motivations for answering questions online, and then grouped them into intrinsic and extrinsic categories. Similarly, Ringel Morris and colleagues [27] identified 10 motivations for answering questions asked on social networking platforms such as Facebook and Twitter: altruism, expertise, properties of the question, nature of the relationship, connecting socially, free time, social capital, obligation, humor, and ego.

The theory of planned behavior provides a conceptual framework for users’ behavioral intentions [28]. According to this theory, personal attitudes toward the behavior, social norms or pressure associated with the behavior, and perceived self-efficacy or behavioral control over the behavior can affect behavioral intentions. Lin [29] combined belief and attitude constructs with intrinsic and extrinsic motivation constructs from the literature to study the motivational structure of knowledge sharing in organizations. Lin’s survey instrument included six constructs: (1) expected organizational rewards, (2) reciprocal benefits, (3) knowledge self-efficacy, (4) enjoyment in helping others, (5) attitudes toward knowledge sharing, and (6) knowledge-sharing intentions.

In addition, groups of contributors to different information systems can be driven by different motivations. Huffaker and Lai [30] examined workers’ motivations for contributing knowledge at IBM. They found that new and younger workers were motivated by achieving recognition from the management, whereas older workers and workers who had worked longer for the company were driven more by altruistic factors, such as a desire to provide mentorship and help the community. Oreg and Nov [31] found that contributors of open source software were mostly motivated by reputation building and self-development, whereas contributors of open source content were driven more by altruism. Moreover, activities in RIMSs that involved targeted sharing of information with specific researchers could be viewed as forms of collaboration (e.g., answering questions). Stvilia and colleagues [32] found that when deciding whether to collaborate with another researcher, researchers gave priority to their intrinsic motivations, such as the quality (i.e., interestingness) of ideas a potential collaborator might have.

One of the indirect indicators of a researcher’s reputation in scholarly communication is her or his scholarly impact, as measured by the number of citations the researcher’s works receives. Hence, researchers’ participation in RIMSs can also be motivated by their desire to increase their citation counts or other alternative metrics of impact. Moed [33] suggested that sharing a publication early by posting its preprint in a preprint database such as arXiv could result in a higher citation count. Other studies (e.g., [34]) showed that publication read counts in RIMSs correlated with citation counts.

### Study design

The design of this study was guided by an analysis of the literature reviewed in the previous section. Existing scales of attitudes and motivations were selected from the literature to guide the design of an interview protocol and a survey questionnaire. The study began by conducting semistructured interviews of 15 researchers between January and July of 2016. The researchers interviewed represented 9 fields of study, 10 institutions, and 5 seniority categories (3 full professors, 3 associate professors, 3 assistant professors, 3 postdocs, and 3 doctoral students).

Two authors independently coded all the interviews by use of an initial coding scheme based on the literature analysis. After comparing, discussing, and resolving any differences in their coding, the two authors formed a new coding scheme with emergent codes and subcategories, and then recoded all the interviews. A detailed account of the findings from the qualitative part of the study is presented elsewhere [17].

The qualitative findings were then used to expand and refine the interview questions and develop a survey instrument. The survey instrument was pretested with 9 participants (4 assistant professors, 2 postdocs, 1 associate professor, and 2 graduate students) representing 6 disciplines (Library and Information Science, Chemistry, Mathematics, Business, Education, and Sports Management) for readability and validity. The finalized survey was distributed online to 1,680 researchers in the fall of 2016 by using Qualtrics survey software. Participants were recruited from 115 universities categorized as Doctoral Universities with Highest Research Activity (DUHRA) in the Carnegie Classification of Institutions of Higher Education [35]. E-mail addresses were collected manually from departmental websites, and participants were contacted individually. When recruiting, an effort was made to obtain a sample stratified by seniority. The following five levels of seniority were used: graduate students, postdocs, assistant professors, associate professors, and full professors.

To be eligible to participate, a participant had to have at least one peer-reviewed publication. The survey instrument was composed of 46 questions. Before participants were interviewed or completed an online survey, they were given a consent form approved by the Human Subjects Committee of Florida State University (FSU HSC Number: 2015.16120). The form contained information about the project, including information about potential risks associated with participation in the data collection. Participants who finished an interview or a survey were e-mailed a $30 Amazon gift card.

## Findings

Participants completed early questions on the survey at higher rates, and 412 participants representing 80 DUHRA universities finished the entire survey, resulting in a response rate of 25%. Participants were approximately evenly distributed by gender and seniority, but there were slightly higher numbers of postdocs and assistant professors. Their disciplines were categorized according to the top five fields of study. However, the categories were distributed unevenly, with the Social Sciences category being the largest and the Humanities category being the smallest (see Table 1).

**Table 1:** Descriptive statistics of the sample.

No.	Field of study	Freq	%	No.	Race	Freq	%	No.	Seniority level	Freq	%	No.	Gender	Freq	%
1	Engineering	75	18.2	1	African American	11	2.7	1	Graduate student	73	17.7	1	Female	180	43.7
2	Humanities	42	10.2	2	Asian	94	22.8	2	Postdoc	101	24.5	2	Male	223	54.1
3	Life Sciences	79	19.2	3	Hispanic or Latino	24	5.8	3	Assistant professor	92	22.3	3	Prefer not to answer	9	2.2
4	Physical Sciences	81	19.7	4	Caucasian	244	59.2	4	Associate professor	72	17.5				
5	Social Sciences	135	32.8	5	Other	13	3.2	5	Full professor	74	18.0				
				6	Prefer not to answer	26	6.3								

Freq = frequency.

### Researchers’ participation in online RIMSs

In the qualitative analysis, the authors used the method of conceptual categorization [36] to categorize participants into three levels of participation in RIMSs based on the research information management tasks reported in the interviews: Readers, Record Managers, and Community Members [17]. Preece and Shneiderman’s Reader to Leader framework [15] was used as a conceptual guide when grouping tasks into levels and determining labels for the groupings. Those classified as Readers might or might not have a profile in a RIMS, but they did not maintain it and did not contribute to the RIMS. They did not answer other members’ questions, and they did not endorse other members for their expertise. Record Managers were defined as researchers who maintained their profiles in a RIMS but who did not contribute to the RIMS beyond that. Finally, Community Members were defined as those who not only maintained their profiles, but also contributed to the RIMS community by answering other members’ questions or endorsing other members’ expertise.

**Fig 1:**
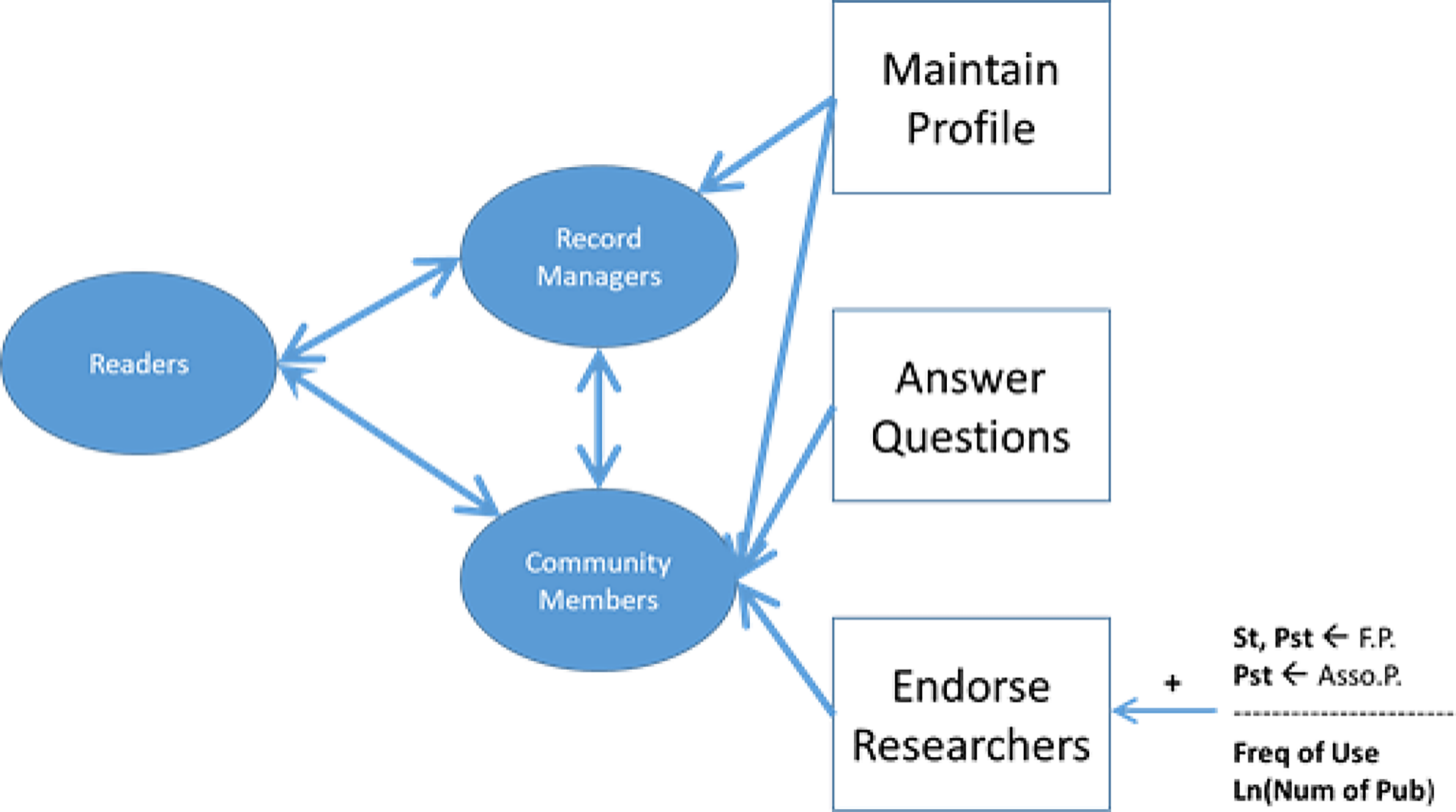
Activities and levels of participation.

Thus, the three participation levels were based on whether researchers completed the following three activities: maintained a RIMS profile, answered questions, and endorsed researchers (see Fig 1). Note that the levels were defined in a progressively cumulative manner. Researchers who belonged to the Record Manager level had to maintain their profile, but they also did not engage in the other two activities: answering questions or endorsing other researchers. On the other hand, researchers who belonged to the Community Member level had to engage in either of those two activities (i.e., answering questions or endorsing researchers) in addition to maintaining their profile.

In the qualitative study, we did not identify any participants acting as Leaders as defined in Preece and Shneiderman’s framework, referring to those who promote participation, mentor novices, and establish community norms and policies. This can be explained by the current design of RIMSs, which do not support that level of participation. In contrast to Wikipedia and other open social knowledge-curation systems, most of the current RIMSs do not enable community self-governance and self-moderation. Furthermore, although researchers may contribute to a RIMS community by performing other activities (e.g., commenting on or reviewing papers uploaded to the RIMS), we used the tasks of answering questions from other researchers and endorsing other researchers for their expertise to differentiate between the Record Manager and Community Member levels. This decision was based on findings from the qualitative study. Researchers most frequently performed the community-level tasks of endorsing others for their expertise and answering questions [17].

The present study also examined the relationships among researchers’ characteristics and each of the three activities. When the binary logistic models of participants’ seniority, discipline, frequency of RIMS use, and number of publications were regressed on whether the participants endorsed other researchers for their expertise, we found that graduate students and postdocs had higher odds of making endorsements than did full professors (χ^2^ = 31.29, *p* = 0.0005, pseudo *R*^2^ = 0.08; *z* > 2.40, *p* < 0.02). Postdocs also had higher odds of making endorsements than did associate professors (*z* = 2.07, *p* = 0.04). In addition, an increase in the number of publications and the frequency of RIMS use increased the odds of researchers endorsing others for their expertise (*z* > 1.90, *p* < 0.05; see Fig 1). No significant differences were found among field of study categories on the odds of endorsing other members for their expertise. Results from the regression analysis for the question-answering and profile-maintenance activities were not statistically significant.

One hundred thirty-eight participants in the survey were categorized as Readers. More than a half of the respondents in the graduate student group were Readers, whereas the proportion of Readers was smallest in the assistant professor group (see Table 2). Concerning the distribution of Readers by field of study, 38% of members of the Humanities category were classified as Readers, the largest proportion compared with other disciplines. The Life Sciences had the smallest proportion (24%; see Table 3). The median number of publications for Readers was 10, and the mean frequency of RIMS use was 3.86. The frequency of RIMS use was measured on a 5-level scale ranging from *Don’t use at all* to *Use several times a day*.

**Table 2:** Levels of participation distributed by seniority.

No.	Seniority level	Readers		Record Managers		Community Members	
		Freq	% of group	Freq	% of group	Freq	% of group
1	Graduate student	41	56	19	26.0	13	17.8
2	Postdoc	27	27	30	29.7	44	43.6
3	Assistant professor	16	17	42	45.7	34	37.0
4	Associate professor	27	38	25	34.7	20	27.8
5	Full professor	27	36	32	43.2	15	20.3

Freq = frequency.

**Table 3:** Levels of participation distributed by field of study.

No.	Discipline category	Readers			Record Managers			Community Members			Total	
		Freq	% of total	% of categ	Freq	% of total	% of categ	Freq	% of total	% of categ	Freq	% of total
1	Engineering	27	6.55	35.99	27	6.55	35.99	21	5.10	28.02	75	18.20
2	Humanities	16	3.88	***38*.*08***	16	3.88	38.08	10	2.43	***23*.*85***	42	10.19
3	Life Sciences	19	4.61	***24*.*05***	23	5.58	***29*.*11***	37	8.98	***46*.*84***	79	19.17
4	Physical Sciences	25	6.07	30.87	33	8.01	***40*.*74***	23	5.58	28.38	81	19.66
5	Social Sciences	51	12.38	37.78	49	11.89	36.28	35	8.50	25.94	135	32.77
	Total	138	33.49		148	35.91		126	30.59		412	100.00

Freq = frequency; % of total = % of total sample; % of categ = % of discipline category.

One hundred forty-eight participants were categorized as Record Managers. Compared with the other seniority categories, the assistant professor category made up the largest proportion of Record Managers. The graduate student category had the smallest proportion (see Table 2). The Physical Sciences category had the largest share of participants (41%) classified as Record Managers, larger than for any of the other discipline categories (see Table 3). The median number of publications for Record Managers was 20, and the mean frequency of RIMS use was 4.0.

The Community Members category comprised 126 participants. In the postdoc category, 44% of the members were categorized as Community Members, the largest proportion compared with any of the other seniority categories. The graduate student category had the smallest proportion of members categorized as Community Members relative to the other seniority categories (see Table 2). According to field of study, the share of Community Members was largest in the Life Sciences category (47%) and smallest in the Humanities category (24%; see Table 3). The median number of publications for Community Members was 20, and the mean frequency of RIMS use was 4.4.

**Fig 2:**
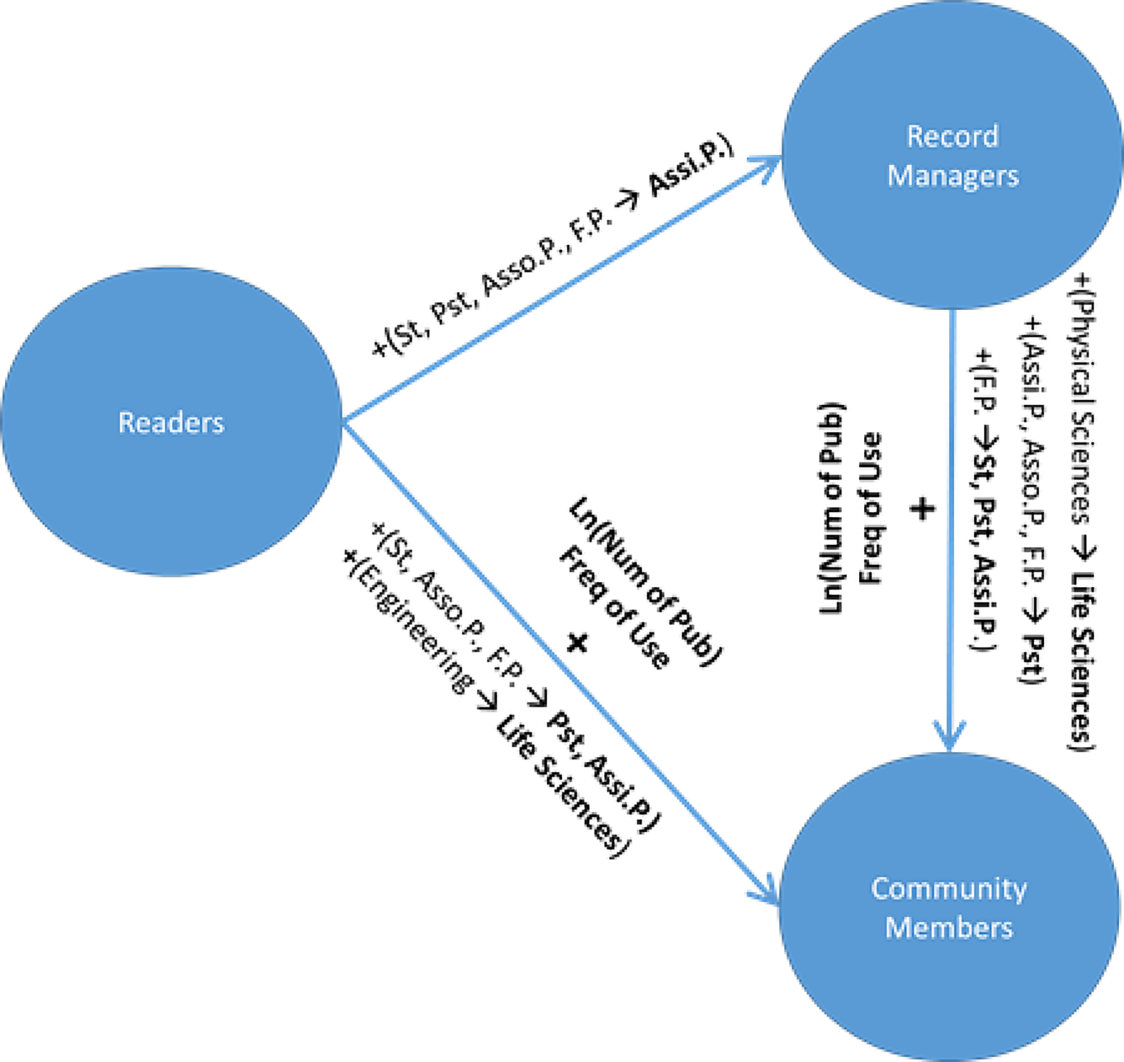
Relationships among researchers’ seniority, frequency of research information management system use, number of publications, and levels of participation. Only statistically significant relationships are included. St. denotes Student; Pst. denotes Postdoc; Assi.P. denotes Assistant Professor; Asso.P denotes Associate Professor, and F.P. denotes Full Professor.

To examine pairwise relationships among the participation levels, we regressed seniority level, field of study, number of publications, and frequency of RIMS use on participation level by using a multinomial logistic regression. The analysis revealed several significant relationships (model fit likelihood ratio: χ^2^ = 76.99; *p* < 0.0001; pseudo *R*^2^ = 0.09). When the Readers level was chosen as the baseline for participation level, switching from the assistant professor to any other seniority category decreased the odds of a researcher being a Record Manager (*z* < –2, *p* < 0.04). The other pairwise relationships among seniority categories were not statistically significant (see Fig 2).

When Readers were compared with Community Members, postdocs and assistant professors had greater odds of being Community Members than did full professors (*z* > 3.75, *p* < 0.001), associate professors (*z* > 2.60, *p* < 0.01), or graduate students (*z* > 2.10, *p* < 0.04). Pairwise relationships between the other seniority categories were not statistically significant. Furthermore, an increase in the number of publications and frequency of RIMS use increased the odds of a researcher being a Community Member rather than a Reader (*z* > 3, *p* < 0.003; see Fig 2). Finally, switching from Engineering to Life Sciences increased the odds of a researcher becoming a Community Member rather than a Reader (*z* = 2.20, *p* = 0.03).

Next, the analysis used the Record Managers level as the baseline for the dependent variable (i.e., participation level) in the regression model. Results showed that switching from the full professor category to the graduate student, postdoc, or assistant professor category increased the odds of a researcher being a Community Member rather than a Record Manager (*z* > 2.10, *p* < 0.04). Likewise, switching from associate professor and assistant professor to postdoc increased the odds of a research being a Community Member rather than a Record Manager (*z* > 2.10, *p* < 0.4).). An increase in the number of publications and frequency of use also increased the odds of a researcher being a Community Member rather than a Record Manager (*z* > 2.20 *p* < 0.03; see Fig 2). Only one statistically significant relationship was found among discipline pairs. Switching from Physical Sciences to Life Sciences increased the odds of a researcher being a Community Member (*z* = 2.49; *p* = 0.02).

### Researchers’ motivations to participate in RIMSs

The three participation levels were based on whether a researcher had completed the following three activities: maintained a RIMS profile, answered questions, and endorsed researchers (see Fig 1). We examined the motivations researchers might have for completing each of the three activities and how they prioritized those motivations. To identify researchers’ motivations for each activity, we presented questions consisting of multiple motivation or attitude statements and asked them to indicate their agreement with each statement on a 7-point Likert scale ranging from *strongly agree* to *strongly disagree*. The study used a dimension reduction technique, factor analysis, to identify their underlying motivational structures for each of the three activities. The identified factors and factor-based models were then used to develop factor-based summated scales of motivations. That is, each scale was composed of the question items that significantly loaded on a factor. Finally, ordered logistic regression was used to identify researchers’ value structure or priorities for the motivation scales for each of the activities.

#### Motivations for maintaining a RIMS profile

Two hundred eighty-one participants indicated that they maintained a profile in a RIMS. The participants were given a closed-ended question consisting of 21 items to identify their reasons for engaging in the profile maintenance activities. Next, we applied a factor analysis with principal components analysis (PCA) to participants’ responses to extract the underlying factors. The component factor matrix was rotated using the Varimax rotation algorithm with Kaiser normalization. A scree plot suggested selecting the first seven factors. Factor loadings of 0.40 and above were identified as significant based on the total number of cases. Variables cross-loaded on more than one factor were removed from the model one by one, and the loadings were recalculated until no such variable was found. The resultant version of the model consisted of 18 variables and 6 factors. Results of a measurement systems analysis (MSA) indicated that each of the variables was higher than 0.59, with the overall MSA equal to 0.75 and the Bartlett test of sphericity significant at the 0.0001 level. The model captured 69% of the total variance of the data.

The study used the extracted factor model to develop six summated scales. The scales were calculated as the mean values of the variables that significantly loaded on a factor. The authors evaluated the internal consistency of the factor scale with Cronbach’s alpha. The alpha values of the scales were greater than 0.70, except for the Support Evaluation scale (see Table 4). Although the alpha value of the Support Evaluation scale was below the generally accepted lower limit of 0.70, it is still considered acceptable for exploratory research [37] and was retained in the analysis with the clear understanding that this scale would need further development.

**Table 4:** Scales of researchers’ motivations for maintaining a research information management system profile.

Scale	*M*	α	Coef(*SE*)^a^	Coef(*SE*)^a^	Coef(*SE*)^a^	Coef(*SE*)^a^	Coef(*SE*)^a^
Scale 5: Share Scholarship • To make my authored content (e.g., papers, data sets, presentations) more findable • To make my authored content more accessible	5.92	0.82	Baseline	2.41(0.17)**	2.26(0.17)**	2.67(0.17)**	3.29(0.18)**
Scale 1: Improve Status • Maintaining my profile is critical to my work • I earn respect as a researcher by maintaining my research profile • I feel that maintaining the quality of my profile improves my status as a researcher • Inaccuracy in my profile can have a negative effect on my status as a researcher	4.51	0.76	−2.41(0.17)**	Baseline	-0.15(0.15)	0.27(0.15)	0.88(0.15)**
Scale 4: Enjoyment • I enjoy maintaining my profile • It feels good to keep my profile current, accurate, and complete	4.34	0.9	−2.26(0.17)**	0.15(0.15)	Baseline	0.41(0.15)*	1.03(0.16)**
Scale 6: Support Evaluation • To correct inaccuracies in my profile introduced by the automated curation • To generate an accurate CV • To help potential employers find me • To help the evaluation of my research productivity and impact	4.28	0.58	−2.67(0.17)**	-0.27(0.15)	−0.41(0.15)*	Baseline	0.62(0.15)**
Scale 2: Quality of Recommendations • To attract students • To receive more accurate recommendations on papers • To receive more accurate recommendations on other researchers	3.68	0.82	−3.29(0.18)**	−0.88(0.15)**	−1.03(0.16)**	−0.62(0.15)**	Baseline
Scale 3: External Pressure • My institution requires me to maintain my profile • My supervisor expects me to maintain my profile • Other researchers encouraged me to maintain my profile	2.43	0.75	−4.92(0.19)**	−2.51(0.16)**	−2.66(0.17)**	−2.25(0.16)**	−1.63(0.16)**

Coef = coefficient; significant relationships are in boldface.

^a^Results of the ordered logistic regression in which the scales were regressed on their values (i.e., summated average ratings; model fit likelihood ratio: χ^2^ = 821.87, *p* < 0.0001, number of observations = 1,644, pseudo *R*^2^ = 0.13).

*p < 0.01.

**p < 0.001.

The ordered logistic analysis of the scales regressed on their values showed that Share Scholarship had significantly higher ratings than did the rest of the scales, followed by Improve Status and Enjoyment. External Pressure had the lowest ratings (see Table 4).

**Fig 3:**
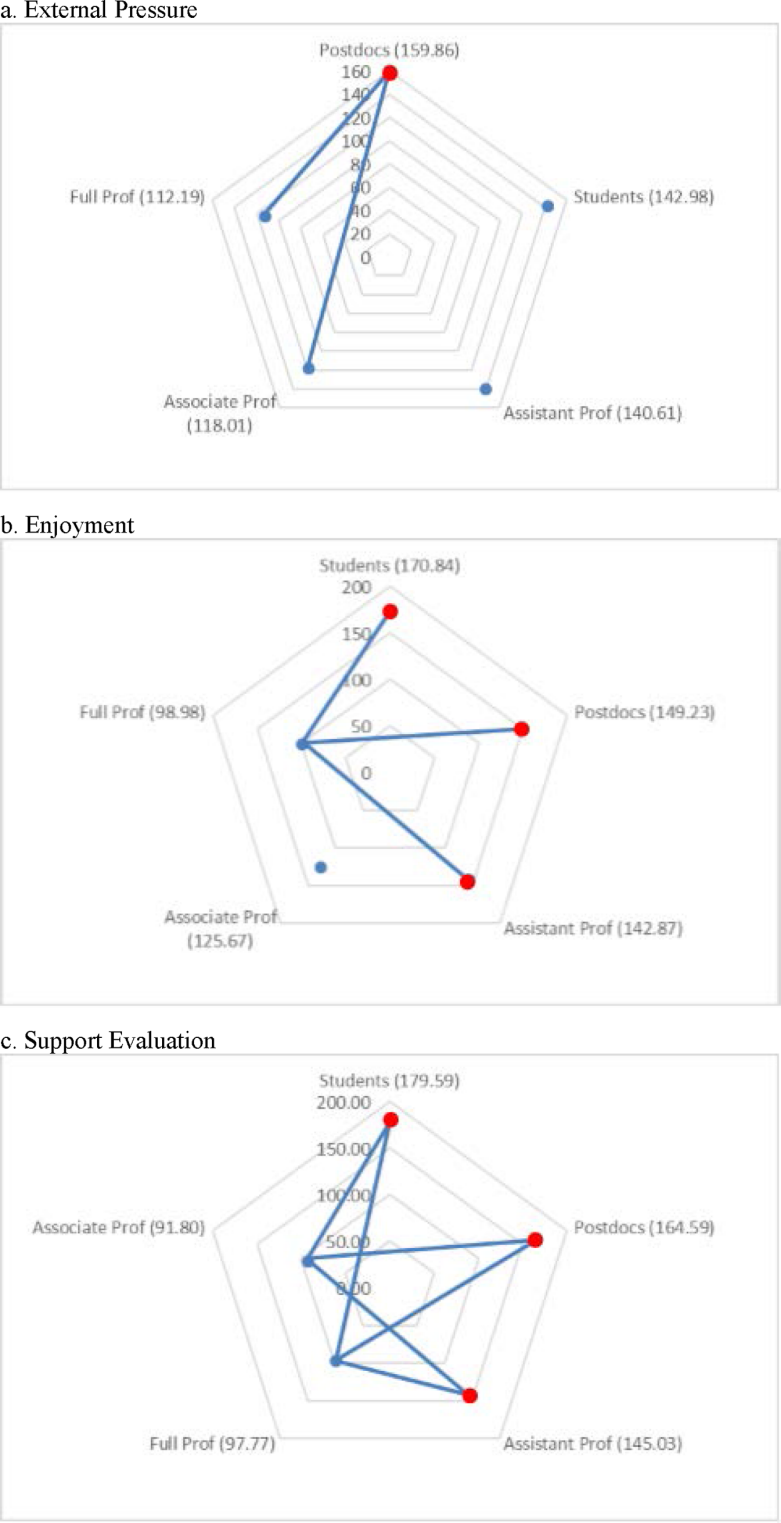
**Pairwise comparison of seniority groups for (a) External Pressure, (b) Enjoyment, and (c) Support Evaluation.** Numbers in parentheses indicate the mean ranks of seniority groups. An edge between the pair of nodes on the graph indicates a statistically significant difference between seniority groups for the motivation scale (*p* < 0.05).

The Kruskal–Wallis omnibus test of the scales on seniority groups revealed significant differences for the External Pressure, Enjoyment, and Support Evaluation scales (χ^2^ = 14.07, *p* = 0.007; χ^2^ = 20.08, *p* = 0.001; χ^2^ = 45.37, *p* = 0.001). The Dunn–Bonferroni tests of post hoc pairwise comparisons indicated that postdocs had significantly higher mean ranks for External Pressure than did full and associate professors. Graduate students, postdocs, and assistant professors had significantly higher mean ranks for the Enjoyment scale than did full professors. Likewise, graduate students, postdocs, and assistant professors had significantly higher mean ranks for the Support Evaluation scale than did associate and full professors (see Fig 3).

**Fig 4:**
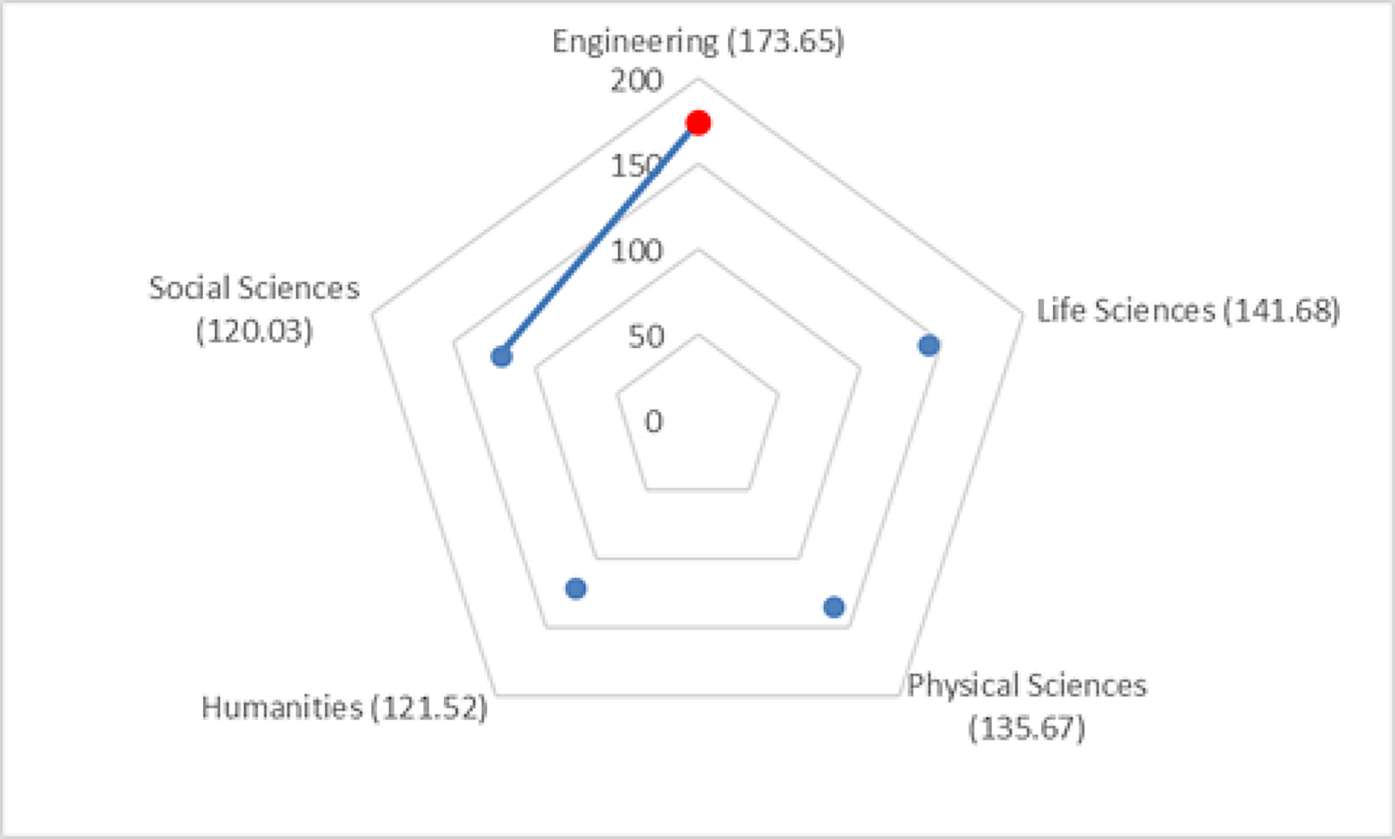
Pairwise comparison of discipline categories for Quality of Recommendations. Numbers in parentheses indicate the mean ranks of discipline categories. An edge between the pair of nodes on the graph indicates a statistically significant difference between discipline categories for the motivation scale (*p* = 0.002).

**Fig 5:**
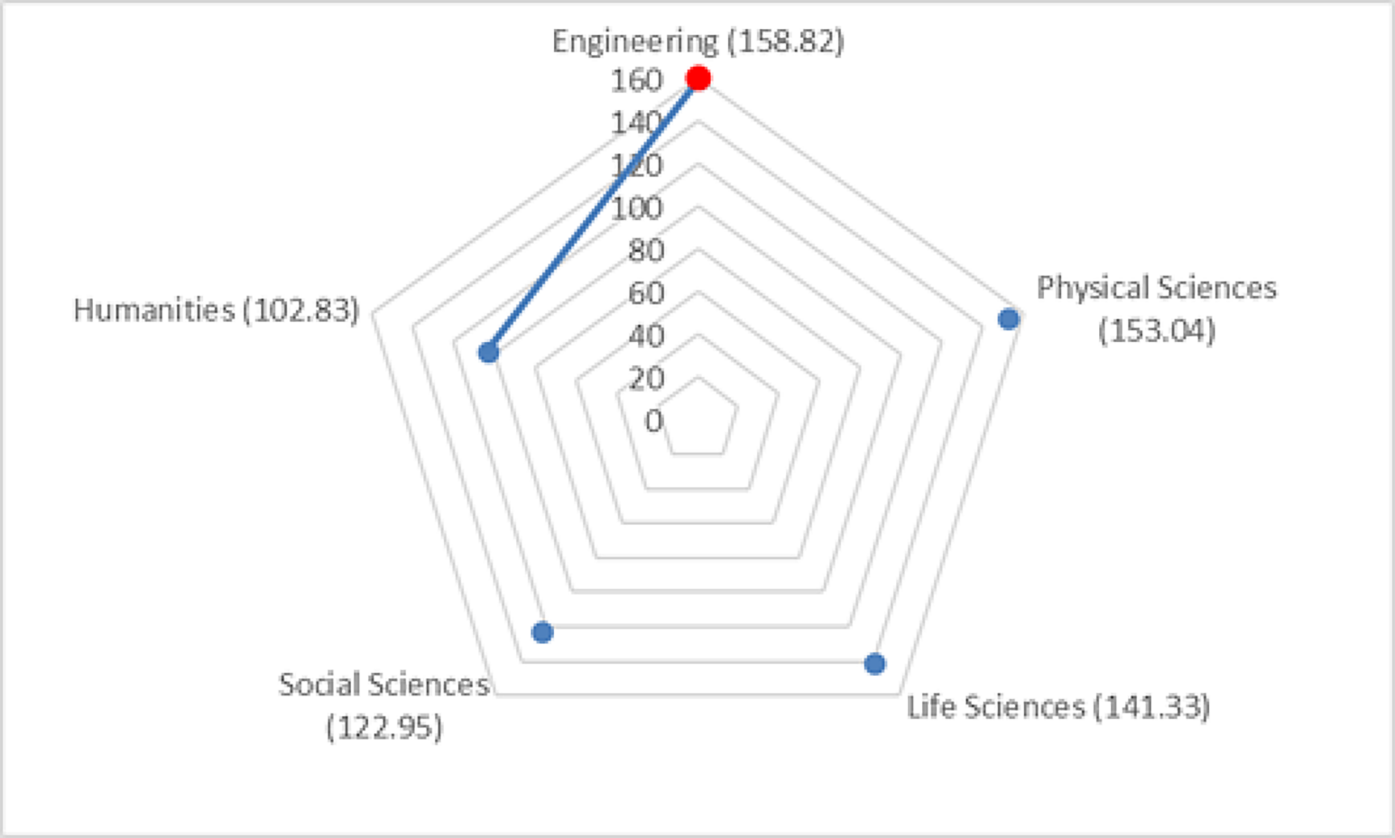
Pairwise comparison of discipline categories for Support Evaluation. Numbers in parentheses indicate the mean ranks of discipline categories. An edge between the pair of nodes on the graph indicates a statistically significant difference between discipline categories for the motivation scale (*p* = 0.036).

The Kruskal–Wallis omnibus test of the scales on discipline categories revealed significant differences for the Quality of Recommendations and Support Evaluation scales (χ^2^ = 15.46, *p* = 0.004; χ^2^ = 13.65, *p* = 0.009). Dunn–Bonferroni tests of post hoc pairwise comparisons in particular indicated that researchers from the Engineering category had significantly higher mean ranks for the Quality of Recommendations scale scores than did researchers from the Social Sciences category (*p* = 0.002; see Fig 4). In addition, researchers from the Engineering category had significantly higher mean ranks for the Support Evaluation scale than did researchers from the Humanities category (*p* = 0.036; see Fig 5).

#### Motivations for answering questions

Next, participants were asked whether the RIMS they used supported asking and answering questions, and whether they answered questions received from other researchers. One hundred fifty-five participants indicated that at least one RIMS they used supported question answering. Fifty-five participants revealed that they answered questions from other members of a RIMS. To identify the underlying structure of researchers’ motivations for answering other members’ questions, the study used a factor analysis. The analysis treated each motivation question item as a variable. The initial model included 16 variables. The study used PCA to extract factors. The component factor matrix was rotated using the Varimax rotation algorithm with Kaiser normalization. A scree plot suggested selecting the first four eigenvalues. Factor loadings of 0.75 and above were identified as significant based on the total number of cases. Variables that had an MSA lower than 0.5 or that were cross-loaded on more than one factor were removed from the model one by one, and the loadings were recalculated until no such variable was found. The resultant version of the model consisted of 10 variables and 4 factors. The MSA of each of the variables was higher than 0.5, with the overall MSA equal to 0.57 and the Bartlett test of sphericity significant at the 0.0001 level. The model captured 80% of the total variance of the data.

The four factors were labeled as follows based on the significant loadings: Build Community Ties, Enjoyment, Expertise, and External Pressure. We used the extracted factor model to develop four summated scales. The scales were calculated as the mean values of variables with significant loadings on a factor. The authors evaluated the internal consistency of the factor scale with Cronbach’s alpha. The alpha values of the scales were greater than 0.85, except for the External Pressure scale (see Table 5). Although the alpha value of the External Pressure scale was below the generally accepted lower limit of 0.70, it was retained in the analysis for theoretical purposes.

**Table 5:** Scales of researchers’ motivations for answering questions from other members of research information management systems.

Scale	*M*	α	Coef(*SE*)^a^	Coef(*SE*)^a^	Coef(*SE*)^a^
Scale 3: Expertise • I am confident in my ability to provide answers that others consider valuable • I have the expertise required to provide valuable answers for others	5.50	0.85	Baseline	1.32(0.35)**	1.50(0.36)**
Scale 1: Build Community Ties • I strengthen ties between other researchers and myself by answering their questions • I expand the scope of my association with other researchers by answering their questions • I expect to receive help from others in answering my questions in return • I believe that my future requests for information/knowledge will be answered	4.86	0.85	−1.32(0.35)**	Baseline	0.18(0.35)
Scale 2: Enjoyment • Answering questions is pleasant • It is fun to answer questions	4.53	0.91	−1.50(0.36)**	**−**0.18(0.35)	Baseline
Scale 4: External Pressure • Other researchers encouraged me to answer the question(s) • I am prompted by the RIMS to answer the question(s)	3.53	0.45	−2.90(0.38)**	−1.58(0.36)**	−1.41(0.36)**

Coef = coefficient; significant relationships are in boldface.

^a^Results of the ordered logistic regression in which scales were regressed on their values (i.e., summated average ratings; model fit likelihood ratio: χ^2^ = 62.89, *p* < 0.0001, number of observations = 220, pseudo *R*^2^ = 0.08).

*p < 0.01

**p < 0.001.

The ordered logistic analysis of the scales regressed on their values showed that Expertise had significantly higher ratings than did the rest of the scales, followed by Build Community Ties and Enjoyment. The External Pressure scale was rated the lowest and was below the neutral value of the scale (see Table 5).

**Fig 6:**
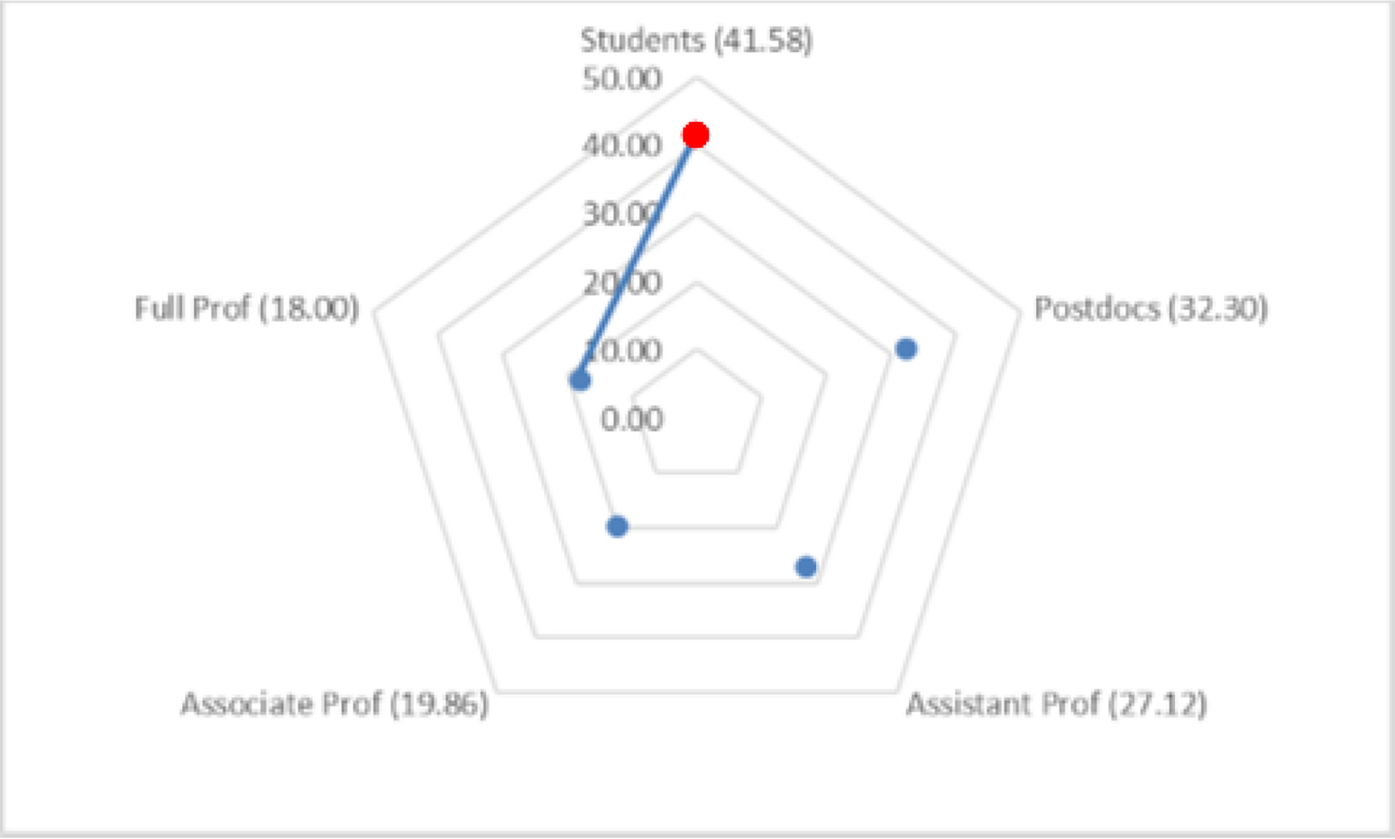
Pairwise comparison of seniority groups for the Build Community Ties motivation scale for answering questions (*p* < 0.05).

The Kruskal–Wallis omnibus test of the question-answering motivation scales on seniority groups revealed significant differences for only Build Community Ties (χ^2^ = 11.57, *p* = 0.02). The Dunn–Bonferroni tests of post hoc pairwise comparisons indicated that graduate students had significantly higher mean ranks for the Build Community Ties scale than did full professors (see Fig 6). A similar analysis of the scales on discipline did not find significant differences.

#### Motivations for endorsing other researchers for their expertise

Two hundred thirty-nine participants indicated that at least one RIMS they used allowed them to endorse other researchers for their expertise. One hundred fifteen of them revealed that they endorsed other RIMS users for their expertise. To identify the underlying structure of researchers’ motivations to endorse other researchers, we used a factor analysis in which each item in the survey question was treated as a variable. The initial model included 10 variables. Principal components analysis was used to extract factors. The component factor matrix was rotated using the Varimax rotation algorithm with Kaiser normalization. A scree plot suggested selecting the first three eigenvalues. Factor loadings of 0.55 and greater were identified as significant based on the total number of cases. Variables with an MSA below 0.5 or that loaded significantly on more than one factor were removed one by one, and factor loadings were recalculated. The resultant version of the model consisted of 8 variables and 3 factors. The MSA of each of the variables was higher than 0.5, with the overall MSA equal to 0.72 and the Bartlett test of sphericity significant at the 0.0001 level. The model captured 82% of the total variance of the data.

The extracted factor model was used to develop three summated scales: Expertise, Enjoyment, and Build Community Ties. The scales were calculated as the average values of variables with significant loadings on a factor. We evaluated the internal consistency of the factor scale with Cronbach’s alpha, and the alpha values of the scales were greater than 0.80 (see Table 6).

**Table 6:** Scales of researchers’ motivations for endorsing other members in a research information management system.

Scale	*M*	α	Coef(*SE*)^a^	Coef(*SE*)^a^
Scale 3: Expertise • I am confident in my knowledge to endorse other researchers for their expertise • I have the knowledge required to endorse other researchers for their expertise	5.41	0.93	Baselin**e**	1.56(0.25)**
Scale 2: Enjoyment • I enjoy endorsing other researchers for their expertise • It feels good to endorse others for their expertise • It is fun to make endorsements	4.47	0.87	−1.56(0.25)**	Baseline
Scale 1: Build Community Ties • I strengthen ties between other researchers and myself by endorsing them for their expertise • I expand the scope of my association with other researchers by endorsing them for their expertise • I expect to receive endorsements for expertise from others in return	4.43	0.84	−1.70(0.25)**	**−**0.14(0.24)

Significant relationships are in boldface.

^a^Results of the ordered logistic regression in which scale name was regressed on scale value (i.e., summated average rating; model fit likelihood ratio: χ^2^ = 57.88, *p* < 0.0001, number of observations = 345, pseudo *R*^2^ = 0.05).

*p < 0.01

**p < 0.001.

Pairwise comparisons of the scales showed that Expertise had significantly higher ratings than did the rest of the scales. Build Community Ties was rated the lowest, although its average value was still above the neutral level of the evaluation scale used for the survey question (i.e., 4; see Table 6).

**Fig 7:**
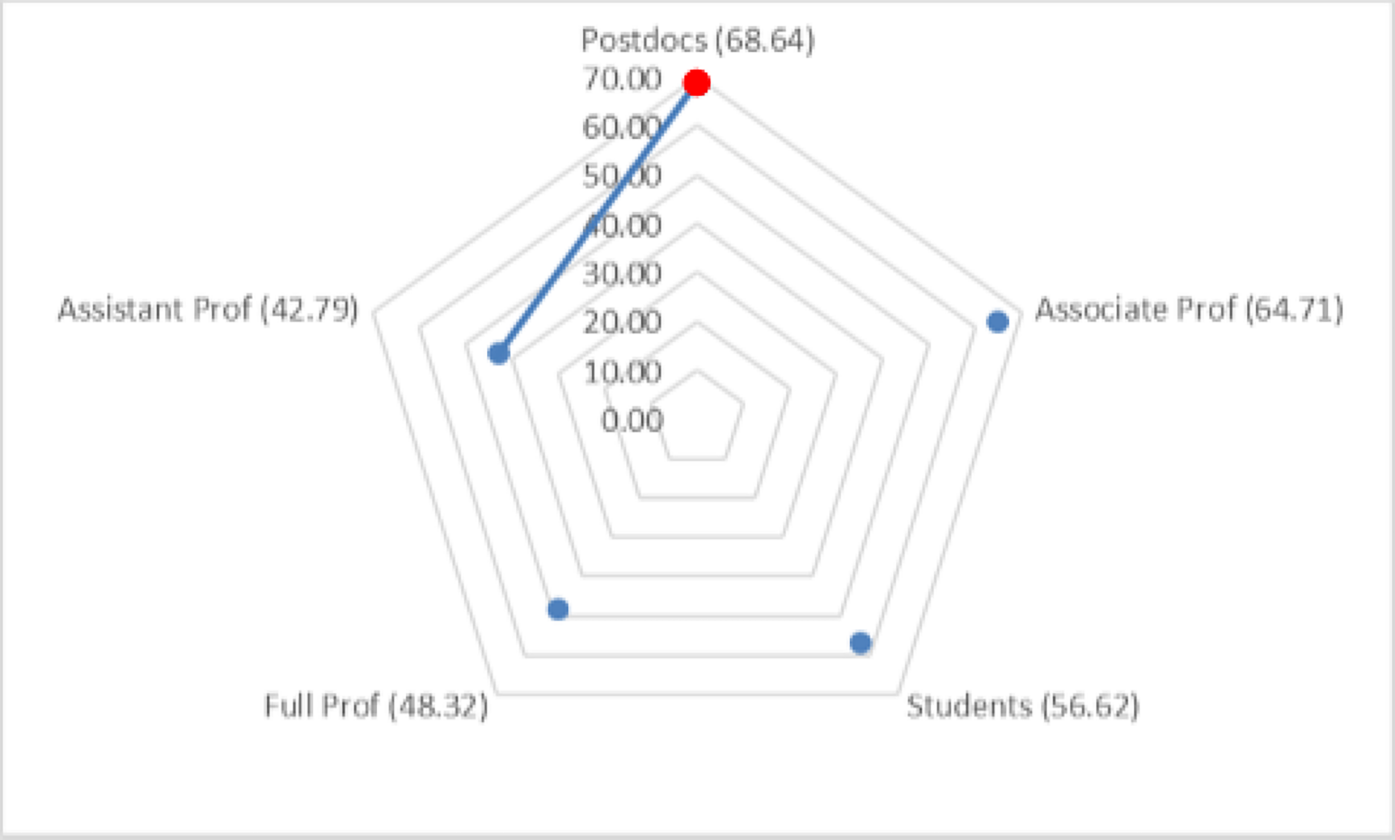
Pairwise comparison of seniority groups for the expertise motivation scale for endorsing other researchers (*p* < 0.05). The numbers in parentheses indicate the mean ranks of seniority groups.

The Kruskal–Wallis omnibus test of the endorsement motivation scales on seniority groups revealed significant differences for the Expertise scale (χ^2^ = 11.57, *p* = 0.02). The Dunn–Bonferroni tests of post hoc pairwise comparisons indicated that postdocs had significantly higher mean ranks for that scale than did assistant professors (see Fig 7). A similar analysis of the scales by discipline did not reveal significant differences.

## Discussion

### Researchers’ participation in online RIMSs

**Fig 8:**
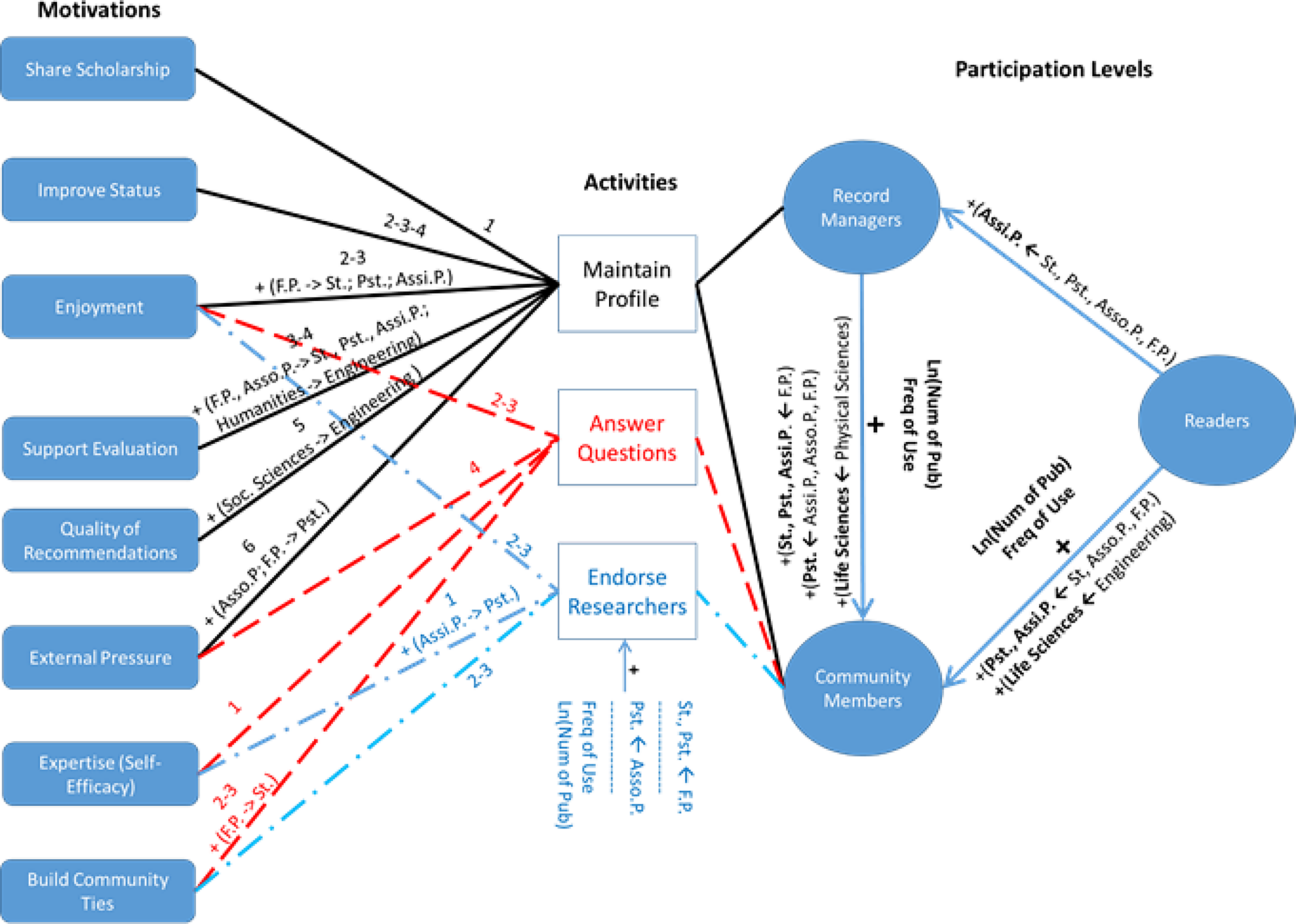
Relationships among activities, motivation scales, and researcher seniority. Numbers represent the ranking of a scale relative to the other scales. More than one number assigned to a motivation scale (e.g., 2-3-4) means that the scale shares the rankings indicated by these numbers with other scales for the activity. St. denotes Student; Pst. denotes Postdoc; Assi.P. denotes Assistant Professor; Asso.P denotes Associate Professor, and F.P. denotes Full Professor.

The first research question examined how researchers participated in RIMSs. We categorized researchers’ participation in RIMSs into three levels: Readers, Record Managers, and Community Members. Graduate students had the greatest share of Readers, whereas assistant professors had the greatest share of Record Managers and postdocs had the greatest share of Community Members (see Table 2). Furthermore, assistant professors had significantly higher odds than did other seniority groups of being Record Managers rather than Readers. Postdocs and assistant professors had significantly higher odds than did the rest of the seniority groups of being Community Members rather than Readers. Likewise, postdocs had a higher probability than did the other seniority categories, except for students, of being Community Members rather than Record Managers (see Fig 8). Thus, overall, assistant professors and postdocs were more engaged in RIMSs as Record Managers and Community Members than were other seniority groups, and postdocs exhibited higher odds than did assistant professors of being Community Members rather than Record Managers. That is, although assistant professors were more focused only on maintaining their RIMS profiles, postdocs were more willing than assistant professors to answer questions or endorse others, in addition to maintaining their RIMS profiles. Finally, as expected, as the frequency of RIMS use and the number of publications increased, the odds of being more engaged in a RIMS as a Record Manager or a Community Member increased (see Fig 2). These findings can be explained by the fact that assistant professors undergo the most frequent formal evaluation as tenure-track faculty and hence have a greater incentive to maintain complete and accurate RIMS profiles. Postdocs, on the other hand, have not yet secured a tenure track position and may be more motivated to cultivate relationships with other members of RIMSs. They may also wish to enhance their visibility though various types of contributions that may not count in the formal models of evaluation applied to assistant professors.

Regarding discipline, the Humanities and Engineering had the greatest shares categorized as Readers and Record Managers. The Physical Sciences had the greatest share categorized as Record Managers, and Community Members made up the largest share of the Life Sciences category (see Table 3). The analysis also showed that life scientists exhibited a significantly higher propensity than did engineers of being Community Members rather than Readers. Furthermore, life scientists had significantly higher odds than did physical scientists of being Community Members rather than Record Managers. These findings about life scientists seem to contradict the findings by Mas-Bleda and colleagues [20] that, at European institutions in general, an online presence in a RIMS is lower in the life sciences than in other disciplines. Our study showed that as Community Members, life scientists were one of the most highly involved disciplinary categories.

The examination of individual activities showed that 55 participants (13%) answered questions from other members of a RIMS, whereas 281 researchers (68%) revealed that they only maintained their RIMS profiles, and 115 researchers (28%) endorsed other members for their expertise. Thus, substantially higher numbers of researchers were willing to endorse other members and maintain their profiles than to answer questions. This result could be explained by the higher cost of answering questions relative to endorsing researchers for their expertise. Another explanation could be that RIMSs might give researchers more opportunities to make endorsements than to answer questions that researchers find interesting and relevant to their research interests. As one participant noted, “The [system’s] UI design encourages [making endorsements]—makes it quick and convenient.” (S71)

Researchers who used RIMSs more often or who had high numbers of publications were more likely to endorse other researchers for their expertise. In addition, postdocs showed greater willingness to endorse other members than did full and associate professors (see Fig 1). This result suggests that junior researchers who might not have as many high-impact publications as senior researchers might assign a higher value to alternative indicators of quality, such as peer endorsements. One postdoctoral participant commented,

I think they deserve it. Maybe I know them. I want other people to know them, to know they are good at this skill. (S11)

In addition, senior researchers who may have higher social capital can be more cautious and nuanced about making endorsements in RIMSs. One full professor commented the following:

My endorsements are NEVER binary. They are context specific, and often take the form of two-page recommendation letters! Endorse: yes/no: I don’t even know what that means. It’s not binary. (S98)

### Researchers’ motivations to participate in RIMSs

The second research question investigated researchers’ motivations to participate in RIMSs. Table 7 provides a summary of the motivation scales developed for the three activities. The numbers indicate the ranking of each motivation scale relative to the other motivation scales for an activity. The question-answering and endorsement activities had similar motivation scales. Both had similar sets of motivation scales and were ranked similarly, although the question-answering activity had one extra scale (External Pressure). Expertise was the top-rated scale for question-answering and endorsement activities. In addition, Enjoyment was common for all three activities and shared the second highest ranking. Build Community Ties shared the second highest ranking with Enjoyment for the question-answering and endorsement activities. Thus, Expertise, Enjoyment, and Build Community Ties were rated higher for the question-answering and endorsement activities compared with other motivations. These motivations can be categorized as intrinsic. Self-determination theory postulates that for a person to be intrinsically motivated, the person must feel competent, inherently autonomous, and related to others and must find the overall activity she or he is performing interesting or pleasant [38].

**Table 7:** Activities and motivations.

Activity	Share Scholarship	Improve Status	Enjoyment	Support Evaluation	Quality of Recommendations	External Pressure	Expertise (Self-Efficacy)	Build Community Ties
Maintain a profile	1	2-3-4	2–3	3–4	5	6		
Answer questions			2–3			4	1	2–3
Endorse researchers			2–3				1	2–3

Numbers represent the ranking of a scale relative to the other scales. More than one number assigned to a motivation scale (e.g., 2-3-4) means that the scale shares the rankings indicated by these numbers with other scales for the activity.

Analysis of the motivations for RIMS profile maintenance identified six motivation scales. The Share Scholarship motivation was ranked significantly higher than the rest of the scales. Research publications and data are the main products of a research activity, and they determine researchers’ standings in their home institutions and peer communities, their promotion, and their tenure [39]. Hence, it was not surprising that the desire to make authored content more findable and accessible was ranked the highest. It is noteworthy that this motivation scale showed no statistically significant difference among different seniority groups.

Quality of Recommendations and External Pressure were ranked significantly lower than the other scales and lower than the neutral value of the Likert scale used in the related survey question. This result suggests that researchers may not consider the quality of recommendations received from RIMSs or social pressure from colleagues or supervisors central to their decision making regarding whether to maintain their RIMS profile. Still, a few participants revealed in their comments that they maintained their RIMS profile because their colleagues did. As one of them explained, she did not want “to appear as an outdated researcher.” It is interesting to note that Enjoyment was evaluated higher than Support Evaluation and above the neutral value. Although Share Scholarship can be linked to external motivation, such as improving the chances of researchers’ having their scholarship cited, some may enjoy the activity. This could relate to the pleasure received from “announcing [their] achievements among peers and friends” (S378) or from enhanced self-worth and satisfaction after providing their peers and the public open access to their scholarship. One participant stated,

I think a lot of it is self-esteem in the profession and wanting to be generous with the scholarship that I’ve worked on and to make it available quite widely. I had a mentor in grad school who really urged us to do everything in the spirit of intellectual generosity, and I’ve tried to practice that whenever possible. I think there are certain [RIM] systems that let you practice intellectual generosity and maybe in a small way. (S13)

Another participant who specialized in South Asian Studies stated that she wanted to “provide open access [to her scholarship] to scholars in the global south” (S70). Hence, in those instances, sharing scholarship could be motivated intrinsically or by integrated regulations. Self-determination theory defines integrated regulation as the type of external motivation that occurs when the objectives of a regulation are fully integrated and aligned with the values and beliefs of the self [38]. These findings also echo the literature on participation in FOSS projects in which altruism and community identification were identified as motivations to contribute [24,25].

Pairwise comparisons of seniority groups for the Support Evaluation scale showed that graduate students, postdocs, and assistant professors had significantly higher ratings for this scale than did full and associate professors. Graduate students, postdocs, and assistant professors also rated the Enjoyment scale significantly higher than did full professors (see Fig 3). These results can be explained by the fact that most full and associate professors have tenure, and hence have less pressure to facilitate their own evaluations.

Pairwise comparisons of discipline categories found engineering researchers to have higher ratings for the Support Evaluation than did humanities researchers. This result could be an indicator of the differences in the scholarly communication and evaluation models used by the two disciplines. Researchers in the humanities favor publishing books and monographs and their evaluation models are often book based, whereas those in engineering fields of study use scholarly communication and evaluation models based more on conference and journal papers [40,41]. Traditionally, books and monographs have received scant coverage in the index databases used in bibliographic analysis of research impact. Hence, compared with the engineering field, the humanities field may rely less on citation-based metrics when evaluating research output and researchers for impact [41]. Furthermore, providing open access to books and monographs has been challenging [42]. Thus, one would expect that the humanities field would rely less than the engineering field on the evaluation models of research impact and the services provided by RIMSs. Similarly, engineers had higher ratings for the Quality of Recommendations scale than did social scientists. One can theorize that researchers in the social sciences might rely more on other means of communication and information sharing (e.g., personal networks) to stay current in their literature than those in engineering. These findings can be used to prioritize RIMS evaluation and recommendation services by discipline. In addition, further research could shed light on alternative models and the means of evaluation and literature monitoring that these disciplines use and could inform the design of RIMS services.

Postdocs rated the External Pressure scale higher than did full and associate professors. This too could be explained by postdocs, as junior researchers, being more dependent on and influenced by their advisors and supervisors, including receiving suggestions on how to promote their scholarship by using RIMSs. On the other hand, senior researchers, by advising their junior colleagues to maintain their profiles, may be motivating themselves to lead by example. One full professor made the following observation:

If I’m advising other colleagues to make sure that their pages are clear or good, it would be kind of bad if mine was not that way. It’s more for peer pressure to myself. (S6)

The analysis of motivations for the question-answering activity identified four scales (see Table 5). Expertise had significantly higher ratings than did the rest of the scales. Answering questions from other researchers was the most cognitively expensive activity among the three activities used in this study to define the RIMS participation levels. Successful completion of the question-answering activity requires information systems to successfully match questions with the competence respondents have. Ardichvili and colleagues [43] found that the fear of providing an inaccurate answer and, as a result, losing face among peers was one of the main barriers to knowledge sharing in an online community of practice. Research communities are competence-based communities [44] that assign the highest priority to the accuracy and reliability of information [45]. Hence, it is not surprising that participants rated feeling competent as the highest motivation for the question-answering activity. It is noteworthy that External Pressure again was rated the lowest and below the neutral value on the Likert scale for the survey question (i.e., 4). Researchers do not appear to be very receptive to prompts received from RIMSs to answer questions posted by other members. One participant noted,

I don’t answer questions posted generally, but if it’s specifically directed to me then I feel I should. (S49)

Enjoyment and Build Community Ties were rated the second highest. No statistically significant difference was found between these two intrinsic motivation scales. Thus, in addition to having the necessary expertise, a researcher might also need to find the question intellectually satisfying and enjoyable to answer, or feel related to or caring for the person who asked the question, or both. One participant explained,

I typically disregard my peers when I can tell they don’t understand my direction. (S289)

Another participant noted that she used a peer’s question “to initiate a higher level discussion for the topic” (S271). These responses echo the findings from the literature that answering a challenging question can provide enjoyment and satisfaction by creating new knowledge, helping others, or simply feeling competent [44,46]. Furthermore, according to the theory of flow, people feel more satisfied when the challenges of tasks they perform match the competence and skill levels they have [13,47].

Pairwise comparisons of the seniority groups for the question-answering motivation scales revealed no significant differences, except for the Build Community Ties scale. Graduate students had the highest mean rank compared with the rest of the seniority groups and had a significantly higher mean rank than did full professors (see Fig 4). One might suggest that senior researchers are already a part of the “invisible college” [48] and have accrued significant social capital [49]. Hence, senior researchers may be less motivated than graduate students to forge new community ties through RIMSs.

The analysis of motivations for the endorsement activity identified only three scales (see Table 6). As with the question-answering activity, the Expertise scale was rated significantly higher than the Enjoyment and Build Community Ties scales. Like the question-answering activity, no statistically significant difference was found between the Enjoyment and Build Community Ties scales. The Kruskal–Wallis test of the scales on seniority groups found significant differences only for Expertise. Postdocs had the highest mean rank for the scale, significantly higher than that for assistant professors (see Fig 5). This last finding is quite intriguing and warrants further investigation. One can only suggest that postdoctoral appointments are typically research-only appointments. On the other hand, in addition to conducting research, assistant professors might be responsible for and have a need to promote their own graduate students and postdoctoral researchers, as well as to build and maintain ties with other colleagues at their institutions or in a greater research community to receive promotion and tenure. Hence, assistant professors may prioritize other motivations, such as group loyalty, when making endorsements. Rashid and colleagues [50] showed that members of an online information system were more willing to make contributions when those contributions benefited smaller groups they were part of rather than the general community. One full professor noted that he made endorsements “to support [his] junior faculty, colleagues, and graduate students” (S9).

## Conclusions

This study examined how researchers participated in RIMSs and their motivations for participation. Profile maintenance, question-answering, and endorsement activities were used to define three cumulatively increasing levels of participation: Readers, Record Managers, and Community Members. Junior researchers were more engaged in RIMSs than were senior researchers. Postdocs had significantly higher odds of endorsing other researchers for skills than did full and associate professors, and they were more likely to be categorized as Community Members. Assistant professors showed a significantly higher propensity to be Record Managers than did any of the other seniority categories. Finally, researchers from the life sciences showed a significantly higher propensity to be Community Members than Readers and Record Managers when compared with researchers from Engineering and the Physical Sciences, respectively.

The study revealed that when performing activities, researchers were motivated by the desire to share scholarship, feel competent, feel a sense of enjoyment, improve their status, and build ties with other members of the community. Moreover, when researchers performed activities that directly benefited other members of a RIMS, they assigned higher priorities to intrinsic motivations, such as perceived self-efficacy, enjoyment, and building community ties. Furthermore, researchers at different stages of their academic careers and from different disciplines ranked some RIM motivations differently. For the RIMS profile maintenance activity, junior researchers enjoyed and were motivated by the desire to support their evaluations more than were senior researchers. In addition, postdocs were more influenced by external pressure than were senior researchers. Regarding discipline, researchers from engineering had higher priorities for the Support Evaluation and Quality of Recommendations scales than did humanities and social sciences researchers, respectively. For the question-answering activity, students were more motivated by the desire to establish community ties than were full professors.

The general model of research participation in RIMSs; the relationships among RIMS activities; the motivation scales for the activities; and activity, seniority, and discipline-specific priorities for the motivations developed in this study provide the foundation for a framework of researcher participation in RIMSs (see Fig 8). The framework can be used by RIMS and institutional repositories to develop tools and design mechanisms to increase researchers’ engagement in RIMSs. The framework can be used to develop motivation measurement instruments, member profile templates, and communication strategies tailored to the different levels of researchers’ engagement, career status, and discipline. Future research will extend the framework by adding models of researchers’ value structure and priorities for RIMS metadata and services.

The study has limitations. The Cronbach’s alpha values of 2 of the 13 motivation scales developed in this study were below the generally accepted lower limit of 0.70. In this study, we retained these two scales (Support Evaluation and External Pressure) in the analyses for theoretical purposes, with a clear understanding that these scales would need further research and development.

## Supporting information

S1 File Survey questions used in this paper. (DOCX)

## References

[1] Cucerzan S. Large-scale named entity disambiguation based on Wikipedia data. In: Proceedings of the 2007 Joint Conference on Empirical Methods in Natural Language Processing and Computational Natural Language Learning. Stroudsburg: Association for Computational Linguistics; 2007. pp. 708–716.

[2] OCLC Research, Task Force on the Registering Researchers. Report of the OCLC Research Task Force on the Registering Researchers. 2014. Available from: http://www.oclc.org/research/themes/research-collections/registering-researchers.html. Cited 17 August 2017.

[3] Wu S, Stvilia B, Lee DJ. Authority control for scientific data: The case of molecular biology. J Libr Metadata. 2012; 12(2–3): 61–82.

[4] DuraSpace. VIVO. 2017. Available from: http://www.vivoweb.org/. Cited 17 August 2017.

[5] Salo D. Name authority control in institutional repositories. Cat Classif Q. 2009; 47(3–4): 249–261.

[6] Heidorn PB. The emerging role of libraries in data curation and e-science. J Lib Adm. 2011; 51(7–8): 662–672.

[7] Lee DJ, Stvilia B. Practices of research data curation in institutional repositories: A qualitative view from repository staff. PLoS ONE. 2017; 12(3): e0173987 doi: 10.1371/journal.pone.0173987 28301533 10.1371/journal.pone.0173987 PMC5354423

[8] Tenopir C, Birch B, Allard S. Academic libraries and research data services In: Current practices and plans for the future; an ACRL white paper. Chicago: Association of College and Research Libraries; 2012.

[9] Giles J. Internet encyclopaedias go head to head. Nature. 2005; 438(7070): 900–901. doi: 10.1038/438900a 16355180 10.1038/438900a

[10] Cosley D, Frankowski D, Terveen L, Riedl J. Using intelligent task routing and contribution review to help communities build artifacts of lasting value. In: Proceedings of the SIGCHI Conference on Human Factors in Computing Systems. New York: ACM; 2006. pp. 1037–1046.

[11] Nov O. What motivates wikipedians? Commun ACM. 2007; 50(11): 60–64.

[12] Stvilia B, Twidale MB, Smith LC, Gasser L. Information quality work organization in Wikipedia. J Assoc Inf Sci Technol. 2008; 59(6): 983–1001.

[13] Kraut RE, Resnick P. Encouraging contribution to online communities In: Kraut RE, Resnick P, editors. Building successful online communities: Evidence-based social design. Cambridge, MA: MIT Press; 2012 pp. 21–76.

[14] Hara N, Sanfilippo MR. Analysis of roles in engaging contentious online discussions in science. J Assoc Inf Sci Technol. 2017; 68(8): 1953–1966.

[15] Preece J, Shneiderman B. The reader-to-leader framework: Motivating technology-mediated social participation. AIS Trans Hum-Comput Interact. 2009; 1(1): 13–32.

[16] Arazy O, Ortega F, Nov O, Yeo L, Balila A. Functional roles and career paths in Wikipedia. In: Proceedings of the 18th ACM Conference on Computer-Supported Cooperative Work & Social Computing. New York: ACM; 2015. pp. 1092–1105.

[17] Wu S, Stvilia B, Lee DJ. Readers, personal record managers, and community members: An exploratory study of researchers' participation in online research information management systems. J Libr Metadata. 2017; 17(2): 57–90. Available from: doi: 10.1080/19386389.2017.1348783

[18] Haustein S, Larivière V. Mendeley as a source of readership by students and postdocs? Evaluating article usage by academic status. In: Proceedings of the IATUL Conferences. West Lafayette, IN: Purdue University; 2014. Available from: http://docs.lib.purdue.edu/iatul/2014/altmetrics/2.

[19] Thelwall M, Kousha K. ResearchGate articles: Age, discipline, audience size, and impact. J Assoc Inf Sci Technol. 2017; 68(2): 468–479.

[20] Mas-Bleda A, Thelwall M, Kousha K, Aguillo IF. Do highly cited researchers successfully use the social web? Scientometrics. 2014; 101(1): 337–356.

[21] Shachaf P, Hara N. Beyond vandalism: Wikipedia trolls. J Inf Sci. 2010; 36(3): 357–370.

[22] Nov O, Naaman M, Ye C. Analysis of participation in an online photo‐sharing community: A multidimensional perspective. J Assoc Inf Sci Technol. 2010; 61(3): 555–566.

[23] Wasko MM, Faraj S. Why should I share? Examining social capital and knowledge contribution in electronic networks of practice. MIS Q. 2005; 29: 35–57.

[24] Hars A, Ou S. Working for free? Motivations for participating in open-source projects. Int J Electron Commer. 2001; 6(3): 25–39.

[25] Lakhani KR, Wolf RG. Why hackers do what they do In: Feller J, Fitzgerald B, Hissam S, Lakhani K, editors. Perspectives on free and open source software. Cambridge, MA: MIT Press; 2005 pp. 3–22.

[26] Raban D, Harper F. Motivations for answering questions online In: Samuel-Azran T, Caspi D, editors. New media and innovative technologies. Mevaseret Zion: Tzivonim Publishing; 2008 pp. 96–110.

[27] Ringel Morris MR, Teevan J, Panovich K. What do people ask their social networks, and why? A survey study of status message Q&A behavior. In: Proceedings of the SIGCHI Conference on Human Factors in Computing Systems; 2010. New York: ACM. pp. 1739–1748.

[28] Ajzen I. The theory of planned behavior: Organizational behavior and human decision processes. 1991; 50(2): 179–211.

[29] Lin HF. Effects of extrinsic and intrinsic motivation on employee knowledge sharing intentions. J Inf Sci. 2007; 33(2): 135–149.

[30] Huffaker D, Lai J. Motivating online expertise-sharing for informal learning: The influence of age and tenure in knowledge organizations. In: Advanced learning technologies: Seventh IEEE International Conference; 2007. Piscataway, NJ: IEEE. pp. 595–599.

[31] Oreg S, Nov O. Exploring motivations for contributing to open source initiatives: The roles of contribution context and personal values. Comput Hum Behav. 2008; 24(5): 2055–2073.

[32] Stvilia B, Hinnant CC, Wu S, Worrall A, Lee DJ, Burnett K, et al Toward collaborator selection and determination of data ownership and publication authorship in research collaborations. Libr Inf Sci Res. 2017; 39(2): 85–97.

[33] Moed HF. The effect of “open access” on citation impact: An analysis of ArXiv’s condensed matter section. J Assoc Inf Sci Technol. 2007; 58(13): 2047–2054.

[34] Thelwall M, Sud P. Mendeley readership counts: An investigation of temporal and disciplinary differences. J Assoc Inf Sci Technol. 2016; 67(12): 3036–3050.

[35] Carnegie Foundation for the Advancement of Teaching. A classification of institutions of higher education. Carnegie Foundation 2017 Available from: http://carnegieclassifications.iu.edu/lookup/custom.php. Cited 17August 2017.

[36] Bailey KD. Typologies and taxonomies: An introduction to classification techniques. Thousand Oaks, CA: Sage; 1994.

[37] Hair JF, Black WC, Babin BJ, Anderson RE, Tatham RL. Multivariate data analysis. Upper Saddle River, NJ: Prentice-Hall; 2005.

[38] Ryan RM, Deci EL. Self-determination theory and the facilitation of intrinsic motivation, social development, and well-being. Am Psychol. 2000; 55(1): 68 11392867 10.1037//0003-066x.55.1.68

[39] Stephan PE. The economics of science. J Econ Lit. 1996; 34(3): 1199–1235.

[40] Moed H. Citation analysis in research evaluation. New York: Springer; 2005.

[41] Wilsdon, J, Allen, L, Belfiore, E, Campbell, P, Curry, S, Hill, S, et al. 2015. The metric tide: Report of the independent review of the role of metrics in research assessment and management. 2015. 10.13140/.2.1.4929.1363.

[42] Crossick G. Monographs and open access. Insights. 2016; 29(1). Available from: https://insights.uksg.org/articles/10.1629/uksg.280/

[43] Ardichvili A, Page V, Wentling T. Motivation and barriers to participation in virtual knowledge-sharing communities of practice. J Knowl Manag. 2003; 7(1): 64–77.

[44] Sharratt M, Usoro A. Understanding knowledge-sharing in online communities of practice. Electron J Knowl Manag. 2003; 1(2): 187–196.

[45] Stvilia B, Hinnant CC, Wu S, Worrall A, Lee DJ, Burnett K, et al Research project tasks, data, and perceptions of data quality in a condensed matter physics community. J Assoc Inf Sci Technol. 2015; 66(2): 246–263.

[46] Wasko MM, Faraj S. “It is what one does”: Why people participate and help others in electronic communities of practice. J Strateg Inf Syst. 2000; 9(2): 155–173.

[47] Csikszentmihalyi M, Rathunde K. The measurement of flow in everyday life: Toward a theory of emergent motivation In: Jacobs JE, Ryan RM, editors. Developmental perspectives on motivation: Nebraska Symposium on Motivation, 1992. Lincoln: University of Nebraska Press; 1993 pp. 57–97. 1340523

[48] Crane D. Invisible colleges: Diffusion of knowledge in scientific communities. Chicago: University of Chicago Press; 1972.

[49] Bourdieu P. The forms of capital In: Richardson G, editor. Handbook of theory and research for the sociology of education. New York: Greenwood; 1986 pp. 241–258.

[50] Rashid AM, Ling K, Tassone RD, Resnick P, Kraut R, Riedl J. Motivating participation by displaying the value of contribution. In: Proceedings of the SIGCHI Conference on Human Factors in Computing Systems; 2006. New York: ACM. pp. 955–958.

