# Supplementary material for: Researchers’ participation in and motivations for engaging with research information management systems
Source: PLoS One. 2018 Feb 23;13(2):e0193459. doi: 10.1371/journal.pone.0193459 (PMC5825153; doi:10.1371/journal.pone.0193459)
Supplement: S1 File — (DOCX) [file pone.0193459.s001.docx]

Survey questions used in this paper

**1.1 How often do you use an online research information management (RIM) system(s) (e.g. Google Scholar, ResearchGate, Academia.edu, your institutional repository) on the average?**

(PICK MOST ACCURATE ANSWER)

Don't use at all

Use less than once a week

Use about once a week

Use several times a week

Use about once a day

Use several times a day

**1.2 Which RIM system(s) have you used? (SELECT ALL THAT APPLY)**

Google Scholar

ResearchGate

Academia.edu

Other (please specify):

**1.3 How many peer-reviewed publications (including conference proceedings) do you have approximately?**

**1.5 Do you have a public research profile in a RIM system(s) (e.g., Google Scholar, ResearchGate, Academia.edu)? Here is an example.**

Yes

No

**1.6 How long have you had a public research pro le in a RIM system(s) (e.g., Google Scholar, ResearchGate, Academia.edu)? If you have pro les in multiple RIM system, use the age of the oldest pro le to answer this question.**

Less than 3 months

Between 3 months and a year

Between 1 year and 2 years

Longer than 2 years

I do not know

1.7 **Does any of the RIM systems you use allow its members to edit and upkeep their profiles (e.g., add/edit information on their affiliation, research interests, or papers)?**

Yes

No

Do not know

**1.8 Do you personally maintain or edit your profile in a RIM system(s)?**

Yes

No

On the next pages you will be given questions with statements with which you may agree or disagree.

Using the 1-7 scale below, please indicate your agreement with each item by selecting the appropriate number on the scale. The 7-point scale is:1 =strongly disagree, 2 = disagree, 3 = slightly disagree, 4 = neither agree nor disagree, 5 =slightly agree, 6 =agree, 7 =strongly agree.

**1.9 Why do you maintain your profile in a RIM system(s)?**

To make my authored content (e.g., papers, datasets, presentations) more findable

To make my authored content more accessible

To correct inaccuracies in my profile introduced by the automated curation of the RIM system

To generate an accurate CV

To help the evaluation of my research productivity and impact

To help potential employers find me

To help potential collaborators find me

To attract students

To receive more accurate recommendations on papers

To receive more accurate recommendations on other researchers

Maintaining my pro le is critical to my work

Maintaining my pro le makes my work more effective

I enjoy maintaining my profile

It feels good to keep my profile current, accurate, and complete

I earn respect as a researcher by maintaining my research profile

I feel that maintaining the quality of my pro le improves my status as a researcher

Inaccuracy in my profile can have a negative effect on my status as a researcher

My institution requires me to maintain my profile

My supervisor expects me to maintain my profile

Other researchers encouraged me to maintain my profile

I am prompted by the RIM system to update my profile

**1.9.1 After reviewing the list above, do any other reasons come to mind (please specify)?**

Reason 1

Reason 2

Reason 3

**1.11 Does any of the RIM systems you use support asking and/or answering questions through a messaging service, Q&A boards, or by other means?**

Yes

No

Do not know

**1.12 Do you answer questions you get through a messaging service OR posted on a Q&A board of any of the RIM systems you use?**

Yes

No

**1.13 Why do you answer other members' questions in a RIM system(s)?**

I enjoy helping others by answering their questions

It feels good to help someone by answering her/his question

Answering questions is pleasant

It is fun to answer questions

I answer the questions I find interesting

I strengthen ties between other researchers and myself by answering their questions

I expand the scope of my association with other researchers by answering their questions

I expect to receive help from others in answering my questions in return

I believe that my future requests for information / knowledge will be answered

I am confident in my ability to provide answers that others consider valuable

I have the expertise required to provide valuable answers for others

I earn respect as a researcher by answering questions from other members

I feel that answering questions improves my status as a researcher

Other researchers encouraged me to answer the question(s)

I am prompted by the RIM system to answer the question(s)

I answer questions to kill time

**1.13.1 After reviewing the list above, do any other reasons come to mind (please specify)?**

Reason 1

Reason 2

Reason 3

**1.15 Does any of the RIM systems you use allow its members to endorse other members for expertise?**

Yes

No

Do not know

**1.16 Do you endorse other members for expertise?**

Yes

No

**1.17 Why do you endorse other researchers for expertise in a RIM system(s)?**

I enjoy endorsing other researchers for expertise

It feels good to endorse others for expertise

It is fun to make endorsements

I strengthen ties between other researchers and myself by endorsing them for expertise

I expand the scope of my association with other researchers by endorsing them for expertise

I expect to receive endorsements for expertise from others in return

I am con dent in my knowledge to endorse other researchers for expertise

I have the knowledge required to endorse other researchers for expertise

I only endorse the researchers whom I know well

I make endorsements to kill time

**1.17.1 After reviewing the list above, do any other reasons come to mind (please specify)?**

Reason 1

Reason 2

Reason 3

**Demographic Information**

**2.1 You are:**

Female

Male

Prefer not to answer

**2.2. You are:**

African American

Asian

Hispanic or Latino

Native American

Caucasian

Other (please specify):

Prefer not to answer

**2.3. What organization(s) are you employed by?**

**2.4. Which of the following positions do you hold at your organization(s)? (SELECT ALL THAT APPLY)**

Administrative (e.g. director, dean)

Academic (e.g. professor, student, instructor)

Research (e.g. research professor, research scientist, research associate, research assistant, postdoc)

Other (please specify):

**2.4.1. What is your current administrative position at your organization? (Select the choice which is the CLOSEST REPRESENTATION of your current position)**

Director

Associate director

Assistant director

Deputy director

Dean

Associate dean

Assistant dean

Other (please specify):

**2.4.2 What is your current academic position at your organization? (Select the choice which is the CLOSEST REPRESENTATION of your current position)**

Full professor

Associate professor

Assistant professor

Visiting professor

Adjunct instructor

Graduate student

Other (please specify):

**2.4.3. What is your current research position at your organization? (Choose the choice which is the CLOSEST REPRESENTATION of your current position)**

Research scientist

Associate research scientist

Assistant research scientist

Postdoctoral research associate

Student research assistant

Technician or programmer Other (please specify):

**2.8 What was the formal discipline of your highest degree?**

**2.9 What are your areas of specialization?**
